# Geographically extensive larval surveys reveal an unexpected scarcity of primary vector mosquitoes in a region of persistent malaria transmission in western Zambia

**DOI:** 10.1186/s13071-020-04540-1

**Published:** 2021-02-01

**Authors:** Dónall Eoin Cross, Chris Thomas, Niall McKeown, Vincent Siaziyu, Amy Healey, Tom Willis, Douglas Singini, Francis Liywalii, Andrew Silumesii, Jacob Sakala, Mark Smith, Mark Macklin, Andy J. Hardy, Paul W. Shaw

**Affiliations:** 1 Institute of Biological, Environmental and Rural Sciences, Aberystwyth University, Aberystwyth, SY23 3FG UK; 2 Lincoln Centre for Water and Planetary Health, School of Geography, College of Science, Think Tank, University of Lincoln, Ruston Way, Lincoln, LN6 7DW UK; 3 Limulunga District Health Office, P.O. Box 910022, Mongu, Zambia; 4 School of Geography, University of Leeds, Leeds, LS2 9JT UK; 5 Provincial Health Office, Western Province, P.O. Box 910022, Mongu, Zambia; 6 School of Public Health and Health Systems, University of Waterloo, Waterloo, ON N2L 3G1 Canada; 7 Ministry of Health, P.O. Box 30205, Lusaka, Zambia; 8 Department of Geography and Earth Sciences, Aberystwyth University, Aberystwyth, SY23 3DB UK; 9 Department of Ichthyology and Fisheries Science, Rhodes University, Grahamstown, South Africa

**Keywords:** Primary vector, Secondary vector, *Anopheles*, Exophagy, Malaria, Residual transmission, Larvae, COI, ITS2

## Abstract

**Background:**

The Barotse floodplains of the upper Zambezi River and its tributaries are a highly dynamic environment, with seasonal flooding and transhumance presenting a shifting mosaic of potential larval habitat and human and livestock blood meals for malaria vector mosquitoes. However, limited entomological surveillance has been undertaken to characterize the vector community in these floodplains and their environs. Such information is necessary as, despite substantial deployment of insecticide-treated nets (ITNs) and indoor residual spraying (IRS) against *Anopheles* vectors, malaria transmission persists across Barotseland in Zambia’s Western Province.

**Methods:**

Geographically extensive larval surveys were undertaken in two health districts along 102 km of transects, at fine spatial resolution, during a dry season and following the peak of the successive wet season. Larvae were sampled within typical *Anopheles* flight range of human settlements and identified through genetic sequencing of cytochrome *c* oxidase I and internal transcribed spacer two regions of mitochondrial and nuclear DNA. This facilitated detailed comparison of taxon-specific abundance patterns between ecological zones differentiated by hydrological controls.

**Results:**

An unexpected paucity of primary vectors was revealed, with *An. gambiae* s.l. and *An. funestus* representing < 2% of 995 sequenced anophelines. Potential secondary vectors predominated in the vector community, primarily *An. coustani* group species and *An. squamosus.* While the distribution of *An. gambiae* s.l. in the study area was highly clustered, secondary vector species were ubiquitous across the landscape in both dry and wet seasons, with some taxon-specific relationships between abundance and ecological zones by season.

**Conclusions:**

The diversity of candidate vector species and their high relative abundance observed across diverse hydro-ecosystems indicate a highly adaptable transmission system, resilient to environmental variation and, potentially, interventions that target only part of the vector community. Larval survey results imply that residual transmission of malaria in Barotseland is being mediated predominantly by secondary vector species, whose known tendencies for crepuscular and outdoor biting renders them largely insensitive to prevalent vector control methods.
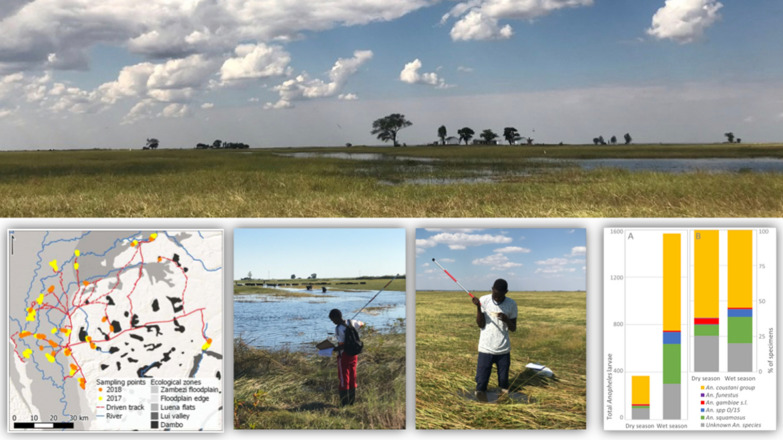

## Background

Without the ambitious global control efforts of the last 20 years, it is estimated that malaria would have killed 995,000 people in 2018; instead, there were 405,000 deaths [1]. Ninety-three percent of malaria cases occur in sub-Saharan Africa [1], where six species of *Anopheles* mosquito are thought to be responsible for 95% of transmission [2]. These primary vector species dominate transmission because of their propensity to obtain blood meals from humans, but the associated endophagic and endophilic behaviors (preference for feeding and resting indoors) render them vulnerable to interventions using insecticide-treated nets (ITNs) and indoor residual spraying (IRS) of insecticides. Together, these vector control methods accounted for 78% of the dramatic global reduction in malaria transmission achieved between 2000 and 2015, during which time an estimated 663 million clinical cases were averted [3].

Traditional indoor-focused interventions may fail to control transmission by exophagic and exophilic mosquitoes, however. This was strikingly demonstrated in the Kilombero Valley in Tanzania, where the introduction of ITNs was less than half as effective at suppressing outdoor-biting *An. arabiensis* mosquitoes than endophagic *An. gambiae sensu stricto* (s.s.) [4]. Many other exophagic and exophilic anopheline mosquito species are typically zoophagic, but those that also feed on humans can consequently play a role as secondary vectors of malaria. Although less efficient than primary vectors [5], secondary vectors can augment or sustain malaria transmission alongside primary vectors and where they are sufficiently abundant may be locally important as main vectors in their own right [2, 5, 6]. Indeed, secondary vectors may be assuming an increasingly significant role in transmission as primary vector populations are suppressed. However, the same outdoor behaviors that make secondary vectors less susceptible to indoor interventions may also permit them to remain largely undetected by surveillance methods dominated by indoor sampling [7, 8]. Lack of detection can be compounded by errors in morphological identification and also by molecular tests seeking to identify primary vectors designated *a priori* from well-established studies in geographically disparate areas [7]. Outdoor collections, although undertaken comparatively rarely, frequently reveal substantially greater vector diversity than could be inferred from prevailing indoor trapping [7].

Successful vector control interventions must be tailored according to vector community composition [9], and therefore accurate species identification is fundamental. DNA-based methods are becoming invaluable in anopheline identification; they have facilitated differentiation between morphologically indistinguishable sibling species (e.g. [10]), highlighted inaccuracies in morphological identification (e.g. [11, 12]) and revealed new or cryptic species (e.g. [13]), even among heavily studied complexes such as *An. gambiae sensu lato* (s.l.) [14]. Sequencing both the mitochondrial DNA (mtDNA) cytochrome *c* oxidase I (COI) gene and nuclear DNA (nDNA) second internal transcribed spacer (ITS2) region has been advocated [70, 71], in recognition of the challenges posed by issues of database coverage [15] and species labelling [7] to universal identification from a single region. Accurate assignment of species—and thus bionomic traits—to specimens allows intervention strategies to target vulnerable behaviors. Subsequent characterization of the spatio-temporal distribution of vector communities can not only facilitate the targeting of interventions, but also monitoring of species’ responses [7]. It is crucial that survey campaigns aiming to describe the entire vector community recognize inherent biases in surveillance approaches so that both endophagic and exophagic populations are sampled. Surveys of larval habitats localized around human habitation avoid such biases by sampling both populations before they become stratified by adult behaviors. Combining DNA-based species identification alongside extensive sampling of larval habitats thus offers considerable, and to date largely unfulfilled, potential to gain a holistic overview of malaria vector communities.

**Fig. 1:**
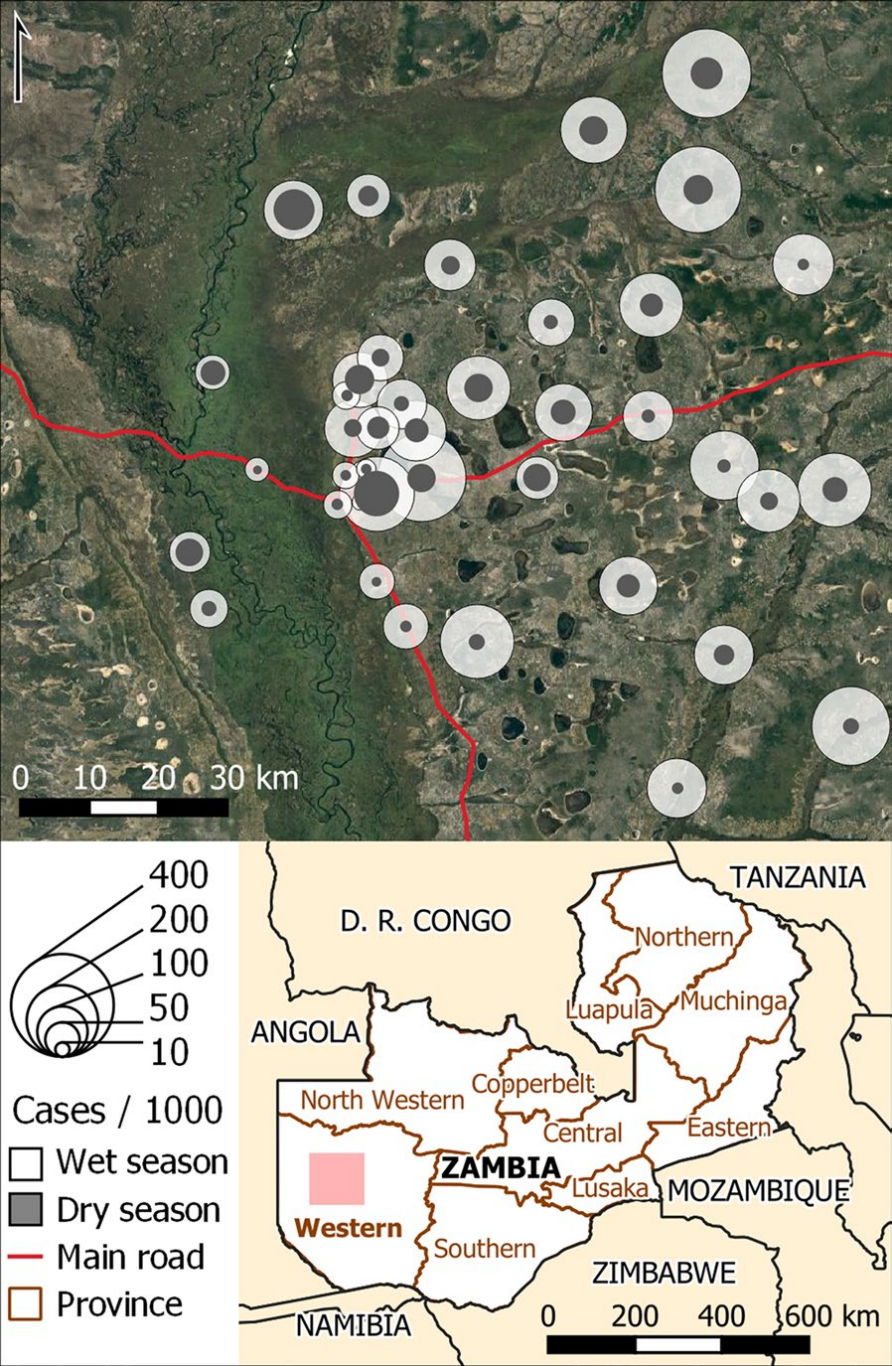
Average seasonal malaria incidence rates in health facilities in Limulunga and Mongu districts, Western Province, Zambia. Rates reported as cases per 1000 population; malaria data provided by Zambian government. Wet season: March–May, averaged 2014–2018; dry season Sept–Nov, 2014–2017. Pink rectangle in inset indicates study extent. Basemap: Google satellite imagery; TerraMetrics

Concerted national malaria control efforts in Zambia have led to a nationwide decline in parasite prevalence among children under 5 from 22 to 9% between 2006 and 2018 [16]. However, rural areas such as much of Western Province continue to suffer disproportionately. Ecologically, the region is dominated by the Barotse floodplain, comprising a vast network of wetlands flooded seasonally by the Zambezi River and its tributaries [17–19], which makes up the area traditionally known as Barotseland. Inundation of the floodplain in the wet season drives an eight-fold increase in the extent of potential mosquito larval habitat [20], and there is evidence of an upward trend in inundation extent over the last decade [17, 21]. Many of the floodplain’s 300,000 human inhabitants [22, 23] practice transhumance in response to the hydrologically dynamic landscape, grazing their cattle in the floodplain in the dry season and shifting them to the uplands when the floodplain becomes inundated [19, 23]. The resultant shifting mosaic of both larval habitat and blood sources for malaria vectors sustains endemic year-round malaria (Fig. 1). The Western Province Health Office has primary-care health facilities distributed throughout the province, including several in the floodplain (Fig. 1). Despite annual application of IRS (30% of households in 2018; [16]) and distribution campaigns tripling the proportion of households with an ITN per sleeping space between 2010 and 2018, malaria parasite prevalence among under-5s in Western Province is currently 10%, twice as high as 2010 levels [16].

Both secondary vectors and exophagic primary vectors can evade indoor interventions and may be implicated in the residual malaria transmission that persists in Barotseland, and so a detailed understanding of the complete vector community of this area is needed to inform future malaria control interventions. A recent molecular-based study of adult specimens collected in eastern Zambia revealed an unexpectedly high diversity of anophelines including primary and secondary vector species, as well as previously undescribed species [12]. The aim of the present study was therefore to employ the same DNA-based species identification methods, in conjunction with a geographically extensive survey of larval habitats, to characterize distribution and abundance of *Anopheles* species assemblages across Barotseland in Zambia’s Western Province. Specific objectives were to identify: candidate primary and secondary vector species present, variation in distribution and abundance of species in the wet and dry seasons and any distinct spatial pattern in species distribution, for example in association with human communities or habitat types.

## Methods

### Sampling strategy

**Fig. 2:**
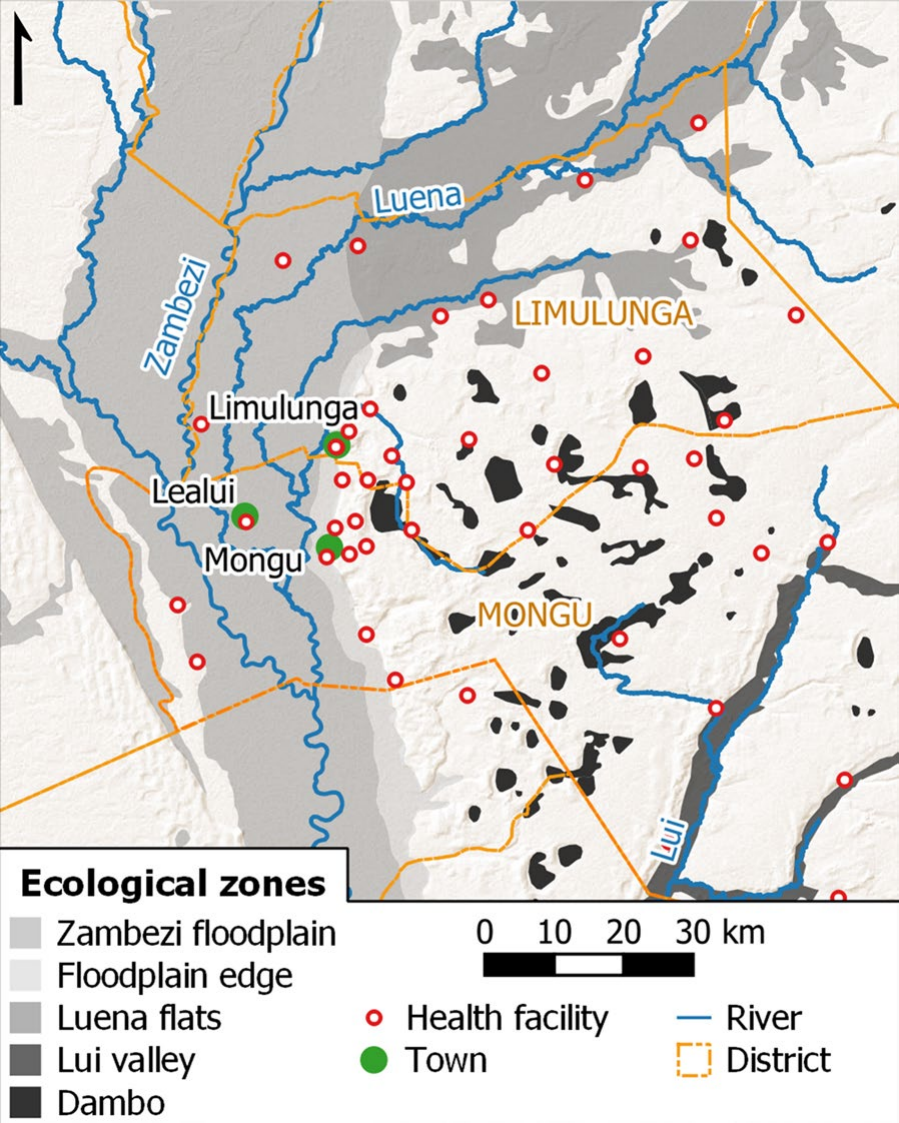
Ecological zones with different hydrological regimes identified in Western Province, Zambia. Health facilities in Limulunga and Mongu districts are shown. Basemap: ESRI Shaded Relief (2020)

The study area in Limulunga and Mongu districts of Western Province of Zambia was partitioned into five broad ecological zones (Fig. 2), defined to represent the different hydrological drivers and vector habitat provision in a typical year, and described below. Larval sampling was undertaken in each zone during the dry season (September–October 2017) and after peak flooding (May–June 2018); Zambezi River discharge in 2018 was the second highest recorded since 1990 (Chalo, C. & Willis, T., 2020, personal communication). The main **Zambezi floodplain** zone is affected predominantly by overbank flow from the Zambezi channel in the wet season, and consists of a mosaic of seasonally flooded grassland, channels and water bodies, interspersed with seasonally occupied human settlements located on mounds known as *mazulu* (see [23]) and one large settlement in the middle of the floodplain. Relatively few water bodies persist in the dry season, being confined to main channels and disconnected features formerly part of active channels [24]. Water bodies in the **floodplain edge** zone, however, persist for much of the year as upland dambos maintain a high water table, which forms seepage zones at the foot of the escarpment (see [23]). This zone, along the eastern edge of the Zambezi floodplain, supports high and year-round human populations because of the agricultural opportunity provided by fertile soils and nutrient-enriched springs [23, 25, 26]. Water bodies in the **Luena flats** zone also persist for much of the year [20], driven largely by the flood regime of the Luena River, resulting in extensive areas of grassland [23] among a highly diffuse anabranching river system [20], which contrasts with the Zambezi; human settlements are restricted to the floodplain edge. East of the Barotse floodplain, aquatic habitats in the narrower **Lui valley** zone are also formed by local, not Zambezi, flooding [26, 27], with human settlements concentrated along the valley edge. The **dambo** zone too is found largely to the east of the Barotse floodplain: dambos are shallow depressions, lined with organic sediments (often peat), which are seasonally or permanently waterlogged, forming an important dry season water source for closely associated human settlements [28, 29].

### Entomological field surveys

We selected two or more health facilities located in representative habitat in each of the ecological zones, with the exception of the Lui valley where only one health facility was chosen because of access constraints. Using knowledge from local health facility staff of current hydrological conditions, two nearby villages in the facility’s catchment area were selected for survey on each sample day. The survey team deployed considerable expertise in sampling *Anopheles* larval habitats gained on previous projects in *An. gambiae* s.s., *An. arabiensis* and *An. funestus* dominated vector systems [30, 31]. Entomological surveys were undertaken along two line transects radiating from each village periphery to characterize the diversity and abundance of anopheline larvae at increasing distances from human habitation [32]. Each transect was up to 1.5 km long, access permitting; approximately 90% of *Anopheles gambiae* s.l. were found to remain within 1.5 km of their larval habitat in similar rural floodplain savannah in The Gambia [32], so a transect of this length was expected to encounter the larval habitats of the majority of anophelines feeding on people in each village. Regular sampling points were located at 100 m intervals along each transect, with additional opportunistic sampling points between the regular ones if water bodies were encountered within 5 m of the transect line.

At each sampling point, an area within a 5 m radius was searched for potential larval habitat, and each water body was geolocated with a GPS handset (Garmin eTrex). A purposive dipping strategy was employed [33–35] to search for mosquito larvae in likely microhabitats, particularly along the water body periphery, among clumps of emergent vegetation and under floating vegetation or debris. Up to 40 dips were taken from each water body with standard 350 ml dippers (BioQuip, USA). The contents of each dip were examined in a white plastic tray, and mosquito larvae were differentiated into anophelines and culicines morphologically and by body position on the water surface. A random sample of up to ten *Anopheles* larvae was collected from each sampling point and stored individually in 95% ethanol for genetic identification.

### Ethical considerations

An ethical approval waiver was provided by the University of Zambia’s Biomedical Research Ethics Committee (Ref 018-08-17). The Barotseland Royal Establishment granted their approval for entomological surveys to be conducted around villages in the study area. District Health Office staff accompanied the field survey team; at the beginning of each day’s fieldwork, the survey team checked in with the nearest health facility and sought permission from village chiefs to undertake fieldwork following introductory discussions.

### Specimen identification

DNA was obtained from larval specimens (crushed with a mounted needle) using a standard CTAB-phenol/chloroform/isoamyl alcohol extraction protocol [36]. Larvae were identified as far as possible to species by sequencing of the mtDNA COI gene and nDNA ITS2 region following protocols outlined in Lobo et al. [12]. ITS2 genotyping was restricted to a subset of individuals from each species or group delineated by COI analyses [12]. Sequence chromatograms were manually trimmed in Chromas (Technelysium Pty Ltd, Australia) and aligned using the Clustal W multiple alignment program [37] implemented in BioEdit [38]. Haplotypes were generated using DNA SP6 [39].

Specimen identities were inferred by a multi-layered consensus of three approaches. First, following Lobo et al. [12], COI sequences were analyzed using the Basic Local Alignment Search Tool nucleotide (BLASTn) to query the National Center for Biotechnology Information (NCBI) nucleotide database (nt) employing the megablast algorithm and applying a standardized cut-off of ≥ 95% similarity. Second, results from BLASTn searches were compared with maximum likelihood phylogenetic trees constructed in MEGA X [40]. Third, ITS2 sequences for the subset of samples based on COI BLASTn results and phylogeny were also analyzed using BLASTn, with the same threshold of ≥ 95% similarity to database accessions.

### Statistical analyses

Fieldwork and laboratory records were compiled in Access (Microsoft) databases and joined to location record shapefiles produced from gpx files from GPS handsets in QGIS 3.10-A Coruña [41]. To enable comparison of the abundance of a species between transect points, the sum total of anophelines recorded in all dips at a transect point was multiplied by the proportion of larvae sampled from that transect point that was molecularly identified as this species. This estimate of the total number of larvae of this species encountered at the transect point was then standardized for the sampling effort by reporting as an encounter rate per ten dips.

Statistical comparisons were undertaken in SPSS [42] on untransformed data [43]; comparisons between dry and wet seasons, or between ecological zones, were made using appropriate parametric (Student’s *t* test; Odds Ratio; ANOVA) or non-parametric tests (Kolmogorov-Zmirnov Z; Kruskal-Wallis *H* with stepwise step-down post-hoc comparisons and adjusted *p *value for multiple comparisons).

#### Results

### Larval habitat sampling

**Fig. 3:**
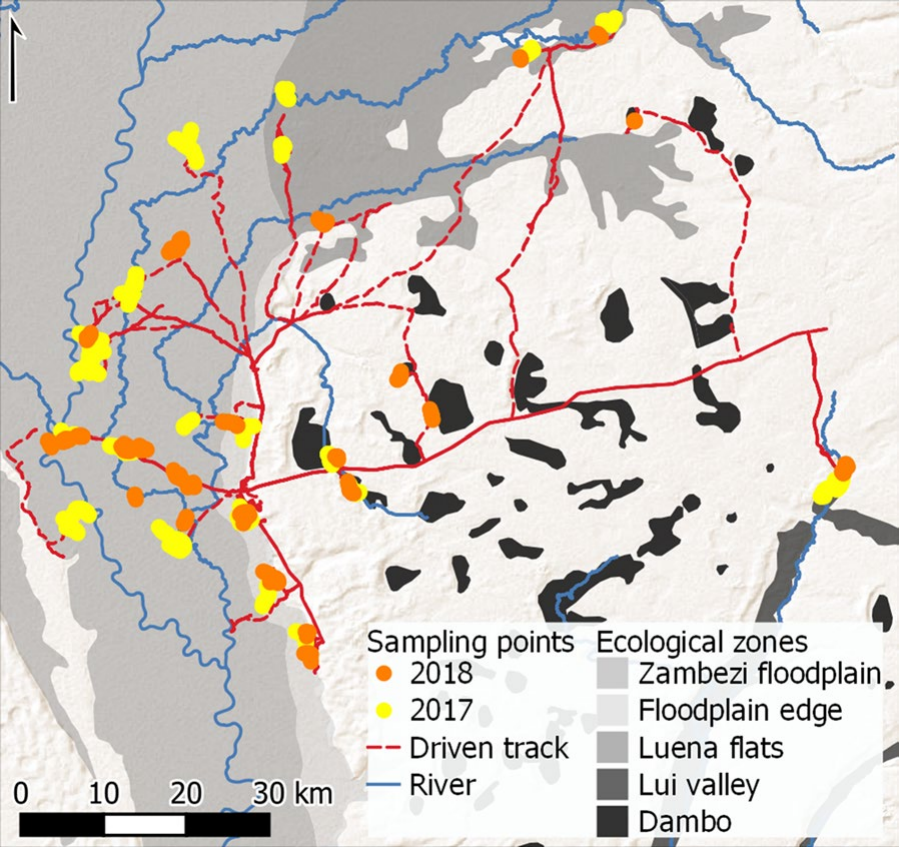
Distribution of sampling points across ecological zones in dry season and wet season entomological surveys. Dry season: Sept–Oct 2017; wet season: May–June 2018, following peak inundation. Sampling undertaken in Limulunga and Mongu districts in Zambia’s Western Province. Basemap: ESRI Shaded Relief (2020)

The distribution of water bodies encountered in sampling in the 2017 dry season and following the peak of the 2018 wet season conformed to expectations for the ecological zones outlined above; see [20] for a detailed spatio-temporal characterization of water bodies across this region of Barotseland during this period. Sampling was undertaken along 70 km of transects in the dry season and 32 km of transects in the wet season (Fig. 3). A similar number of transects was completed in both seasons, but difficult access to field study sites and the frequent presence of impassably deep water resulted in a lower average transect length in the wet season (mean ± standard deviation (SD): dry season 1316 ± 309 m, *n* = 53; wet season 666 ± 317 m, *n* = 48; Student’s *t* test 10.321, *df* 99, *p* < 0.001). Consequently, there were 18 transect points on average along dry season transects (SD 3.2), while wet season transects averaged nine transect points (SD 3.3). Water was encountered at a significantly higher proportion of transect points in the wet season than in the dry season (80.1 and 44.6%, respectively; odds ratio [OR] 5.01, 95% CI 3.823–6.566; *p* < 0.001 [44]). The presence of larger water bodies in the wet season necessitated a larger number of dips per transect point to ensure representative sampling of the higher level of variance expected across such water bodies (mean dips ± SD: wet season 23 ± 5.4; dry season 13 ± 4.7).

A significantly higher proportion of transect points where water was found (‘wet transect points’) contained mosquito larvae (including anophelines) in the wet season (95%) than in the dry season (62.3%; OR 11.49, CI 6.781–19.47, *p* < 0.001). A similar pattern was observed in anopheline distribution, whereby 85% of wet transect points in the wet season were positive for anophelines compared to 43.3% in the dry season (OR 7.405, CI 5.184–10.578, *p* < 0.001). Approximately one third of mosquito larvae encountered were field-identified as anophelines (3045 of 11504 larvae; *n* = 13,335 dips), and the average number per ten dips in wet season surveys was double that of the dry season average (2.8 ± 9.13 and 1.4 ± 6.47, respectively; Kolmogorov-Smirnov *Z* 4.974, *p* < 0.001).

### Genetic identification of larvae

**Fig. 4:**
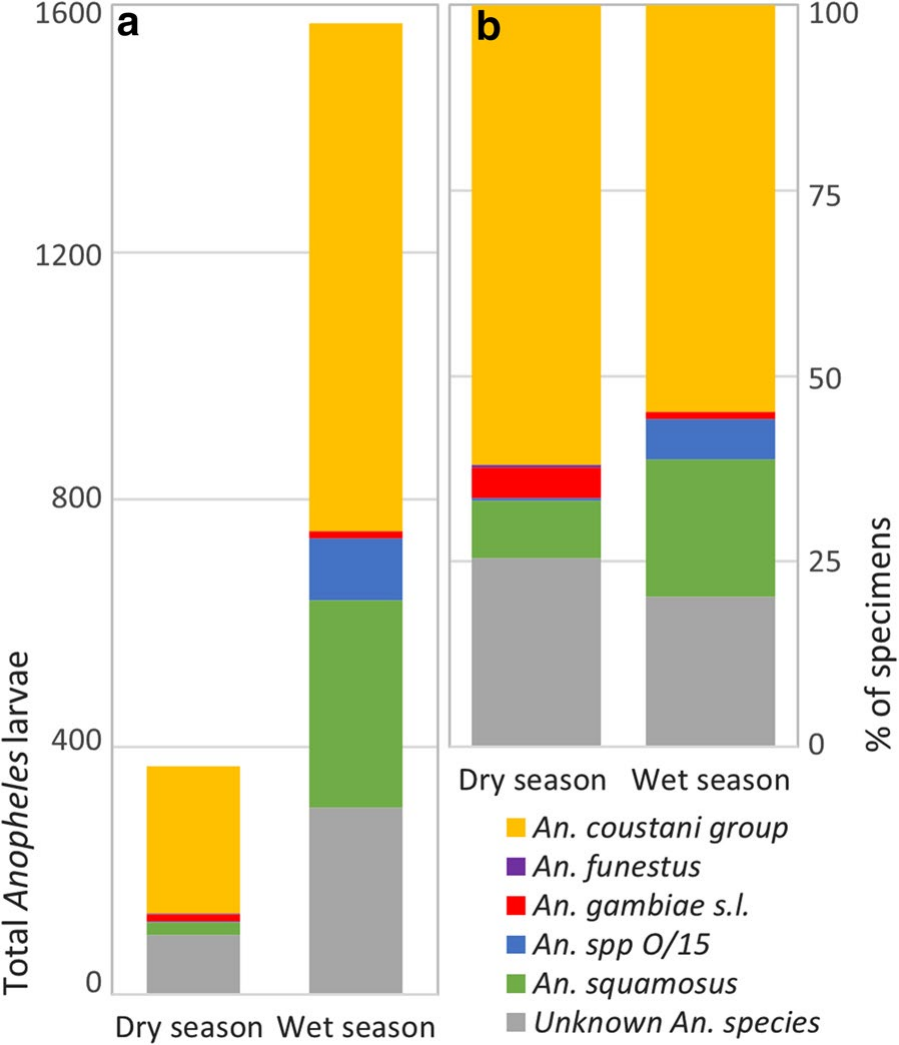
Abundance and species composition of larval *Anopheles* communities in dry and wet seasons, western Zambia. **a** Estimated total abundance of *Anopheles* species (/groups), calculated by applying species proportions from a subset of sampled larvae (**b**) to the total number of surveyed larvae per transect point and summing for all transect points. **b** Composition of specimens identified from cytochrome c oxidase I and/or internal transcribed spacer region 2 DNA sequences. “Unknown *An*. species” assigned to specimens whose alignment with NCBI nt accessions fell below the 95% identity threshold, but most closely matched to anopheline sequences. Sampling undertaken in Limulunga and Mongu districts, Western Province; dry season: Sept–Oct 2017; wet season: May–June 2018. Basemap: ESRI Shaded Relief (2020)

A total of 1034 specimens were collected for individual DNA analysis (of the 3045 encountered). A 318 bp fragment of the COI region was aligned across 903 specimens, with sequences resolving 413 haplotypes. BLASTn searches of the NCBI nt database revealed matches at ≥ 95% identity for 83% (*n* = 340) of these haplotypes, corresponding to 81% of specimens. Thirty-nine specimens were identified as culicines (non-anophelines). The above-threshold BLASTn hits to anophelines could be partitioned into five groups (Table 1). The majority of haplotypes (*n* = 203) and individuals (*n* = 475) were assigned to the *An. coustani* group (> 95% identity with at least one of *An. coustani, An. conferre (cf.) coustani 1* [12]*, An. cf. coustani 2* [12]*, An. tenebrosus* or *An. ziemanni*); 157 specimens were identified as *An. squamosus* and 42 as *An. species* O/15 [11]. Only 14 were identified as *An. gambiae* s.l. (> 95% identity with at least one of *An. arabiensis, An. coluzzii, An. fontenillei* or *An. gambiae* s.s.) and 1 as *An. funestus* s.s. A group of 175 specimens returned NCBI nt matches below the 95% sequence similarity threshold, but where the closest matches on the database were all to anopheline species. These individuals do not match with sequence similarity of > 95% to any of the taxa identified by Lobo et al. [12] or St Laurent et al. [11], despite 100% query coverage with longer sequences deposited by these studies in NCBI nt. Individuals in this group were therefore categorized as “Unknown *Anopheles* species” (Table 1 and Fig. 4; further details in Additional file 1). A maximum parsimony phylogenetic tree of haplotypes from this study and reference sequences from recent studies in Zambia and Kenya [11, 12] supported the COI-assigned identities (Additional file 2).

**Table 1:** Species and species-group identities assigned to *Anopheles* larvae based on mitochondrial and nuclear DNA sequences

Final ID	Potential member species of group/complex (if applicable)	COI-assigned specimens (haplotypes)	Additional ITS2-assigned specimens	Dry season	Wet season	Total
*An. coustani* group	*An. coustani* s.s., *An. cf. coustani* 1, *An. cf. coustani* 2, *An. tenebrosus*, *An. ziemanni*	475 (203)	88	153	410	563
*An. funestus*	–	1 (1)	0	1	0	1
*An. gambiae* s.l.	*An. arabiensis*, *An. gambiae* s.s.	14 (6)	3	10	7	17
*An. species* O/15	–	42 (22)	0	1	41	42
*An. squamosus*	–	157 (79)	0	19	138	157
Unknown *An*. species	–	175 (71)	52	63	152	215
	Total	864 (382)	143	247	748	995

Identities inferred from above-threshold matches to cytochrome c oxidase I and/or internal transcribed spacer region 2 sequences on the National Center for Biotechnology Information nucleotide database (NCBI nt). Sampling undertaken in dry season (Sept–Oct 2017) and wet season (May–June 2018) in Limulunga and Mongu Districts, Western Province, Zambia. Of 3045 field-identified anophelines encountered, 1034 specimens were collected for genetic identification; 39 were subsequently identified as culicines and not reported in this table. “Unknown *An*. species” indicates below-threshold (<95%) matches to *Anopheles* sequences in NCBI nt; 12 specimens assigned to this group from COI sequences were subsequently re-assigned to other taxa based on ITS2 sequences

COI identities were supported by representative ITS2 sequences for the *An. coustani* group (matches with *An. cf. coustani 1*, *An. coustani*) and *An. funestus*. ITS2 sequences supporting COI-assigned *An. gambiae* s.l. specimens indicated the presence of *An. arabiensis* (60%) and *An. gambiae* s.s. (40%); none of these specimensʼ ITS2 sequences demonstrated a match with *An. coluzzii* or *An. fontenillei*, and neither have been found in Zambia, although the former is known to occur in neighboring countries [45]. ITS2 sequences from specimens identified from COI sequences as *An. species O/15* failed to return a match with ≥ 95% identity to any accession. Among 27 ITS2 sequences from COI-assigned *An. squamosus* specimens, 56% matched with *An. cf. coustani 1*, 11% equally with *An. species O/15* and *An. cf. coustani 1,* 7% matched with *An. pharoensis*, while the remainder were unmatched at ≥ 95% identity, likely reflecting the paucity of ITS2 accessions for *An. squamosus* on NCBI nt (*n* = 3). In cases where individuals exhibited above-threshold COI assignments with apparently discordant ITS2 scores, we presently assign identification based on their COI identity and discuss the potential reasons for these discordances and implication for final species proportions.

The ITS2 sequencing also included some individuals without COI sequences (*n* = 131) and some assigned to the unknown anopheline category based on COI sequences (*n* = 12). ITS2 sequences for these specimens permitted additional assignments to the *An. coustani* group (88 individuals), *An gambiae* s.l. (3 individuals; *An. arabiensis* (*n* = 1) and *An. gambiae* s.s. (*n* = 2)) and the unknown anopheline category (52 individuals which aligned at < 95% similarity with NCBI nt *Anopheles* sequences; details in Additional file 1). Final species totals are reported in Table 1 and proportions observed in the dry and wet seasons shown in Fig. 4.

The majority of *Anopheles* larvae identified by sequencing in both the dry season (*n* = 247) and wet season (*n* = 748) were identified as *An. coustani* group (62 and 55%, respectively; Fig. 4). Species considered primary malaria vectors were rare in the sample; a single *An. funestus* s.s. specimen was found in the wet season, while very small numbers of *An. gambiae* s.l. were found in both the dry (*n* = 10) and wet (*n* = 7) seasons. *An. squamosus* made up a substantially greater proportion of sequence-identified samples in the wet (18%) than in the dry season (8%). *An. species O/15* was represented by a single individual in the dry season, but made up 5% of sequence-identified specimens in the wet season. Between a fifth and a quarter of sequences did not align with > 95% identity to any sequence on the NCBI nt database, and were designated unknown *An.* species (dry season: 26%; wet season: 20%).

### Spatial distribution of species

Due to significant differences between ecological zones in the number of dips taken per transect point, comparisons between ecological zones are made on a standardized metric, whereby average total abundance values per transect point are presented per ten dips, i.e. with constant sampling effort.

There was no difference in the standardized mean total *Anopheles* larvae per transect point between ecological zones in the dry season (*F *= 0.984, *df* 4, *p* = 0.416). In the wet season, *Anopheles* larvae distribution differed significantly between ecological zones (Kruskal-Wallis *H *= 24.938, *df* 4, *p* < 0.001); fewer *Anopheles* larvae were found per transect point in Zambezi floodplain and floodplain edge habitats than in the Luena and Lui catchments and in dambos (stepwise step-down comparisons; adjusted (adj) *p* < 0.05; Fig. 6a).

**Fig. 5:**
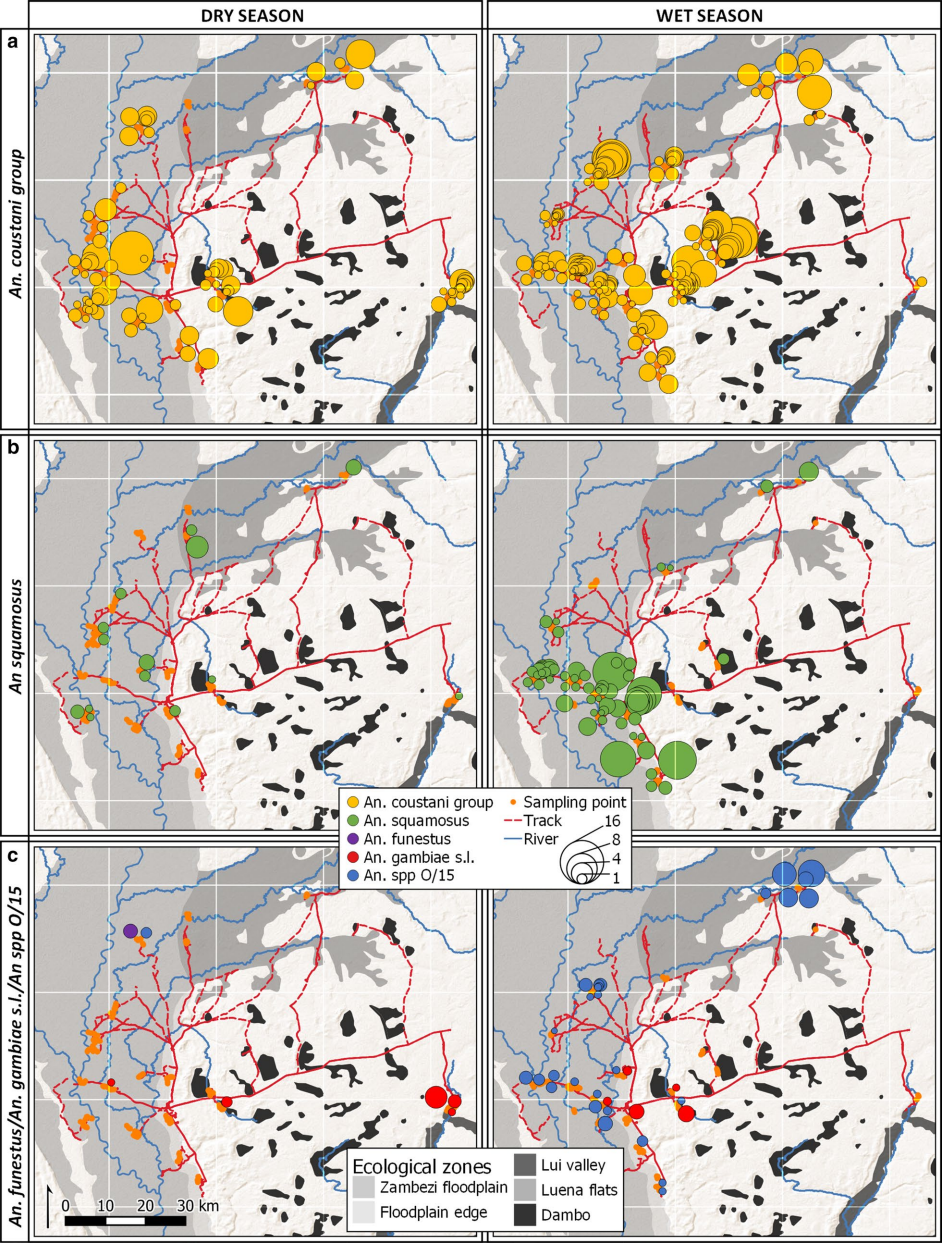
Distribution of *Anopheles* larvae across ecological zones in dry and wet seasons, western Zambia. **a** *An. coustani* group; **b** *An. squamosus*; **c** *An. funestus*, *An. gambiae* s.l. and *An. spp O/15*. Dry season sampling undertaken Sept–Oct 2017 (left column); wet season sampling May–June 2018 after peak inundation (right column). Symbol area proportional to total larvae per transect point (product of total anopheline count and species proportion in subsample), standardized per ten dips. Basemap: ESRI Shaded Relief (2020)

**Fig. 6:**
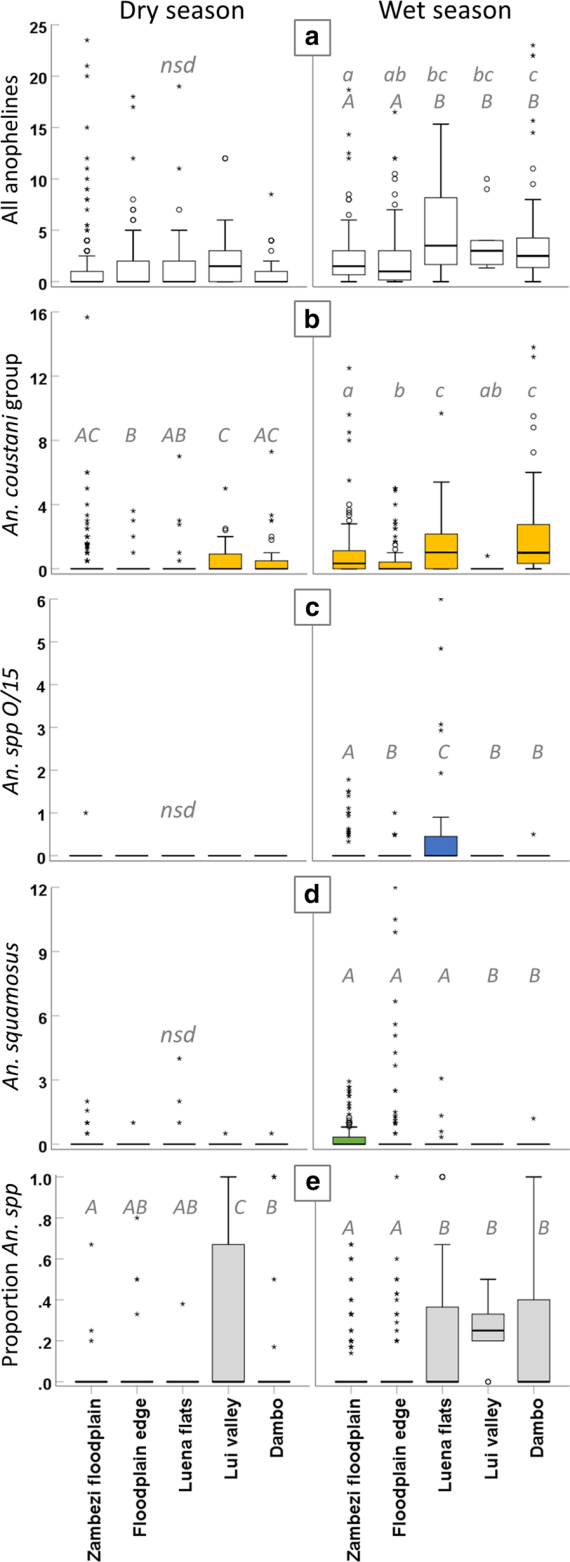
Average abundance of *Anopheles* larvae across ecological zones in dry and wet seasons, western Zambia. Boxes indicate the interquartile range (IQ) of total values per transect point, with median plotted as bold line; whiskers extend to minimum and maximum values within 1.5 times the IQ; outliers (values within 1.5 to 3 times the IQ) are indicated by circles, and extreme values (> 3 times the IQ) by asterisks. **a** total anopheline larvae per ten dips; **b** total *An. coustani* group larvae; **c** total *An. spp O/15*; **d** total *An. squamosus*; **e** proportion of unknown anophelines per transect point. Taxon-specific totals (**b–d**) are the product of total anopheline count and species proportion in the subsample from each transect point, standardized per ten dips. Within panels, letters in italics denote statistical comparisons. Ecological zones that do not share a letter within a panel are significantly different. Lower case letters refer to median values (independent-samples median test); upper case letters refer to distribution (Kruskal-Wallis test); stepwise step-down comparisons with adjusted *p*-value for multiple comparisons. *Nsd* indicates no significant differences within panel. Dry season sampling undertaken Sept–Oct 2017 (left column); wet season sampling May–June 2018 after peak inundation (right column)

In the dry season, *An. coustani* group larvae were distributed ubiquitously across all ecological zones (Fig. 5a), although significantly fewer were found in floodplain edge habitats than in all others except the Luena flats; abundance in the Lui valley was higher than in the Luena flats (Kruskal-Wallis *H* = 29.435, *df* 4, *p* < 0.001; stepwise step-down comparisons with adj *p* < 0.05 Fig. 6b). *An. squamosus* larvae were present in all ecological zones (Fig. 5b), and there was no significant difference between zones in the average standardized total per transect point (*H *= 3.216, *df* 4, *p* = 0.522; Fig. 6d). Only ten *An. gambiae* s.l. larvae were encountered in the dry season, in a range of ecological zones, although eight of these were found in three adjacent transect points within 140 m in the Lui valley. Another *An. gambiae* s.l. was found in a pool adjacent to a tributary of the Zambezi in the floodplain, and another in a dambo immediately east of Mongu (Fig. 5c). One *An. funestus* larva was found in a pool on a transect in the Luena flats, and a single *An. species O/15* larva was found on the neighboring transect from the same village. The proportion of unknown anophelines was lowest in the floodplain and significantly higher in the Lui valley ecological zone than in all other zones (*H = *86.214, *df* 4, *p* < 0.001; stepwise step-down comparisons, adj *p* < 0.05; Fig. 6e).

After the peak of the 2018 wet season, *An. coustani* group mosquitoes were again ubiquitous across the sampled area (Fig. 5a), but median abundance was significantly higher in the dambo and Luena floodplain ecological zones than in the floodplain edge and Lui valley zones (*H* = 48.563, *df* 4, *p* < 0.001; stepwise step-down comparisons, adj *p* < 0.05; Fig. 6b). In contrast to the dry season, distributions of *An. squamosus* abundance in the Luena and Zambezi floodplains and along the floodplain edge ranked significantly higher than those of dambo habitats (*H *= 19.227, *df* 4, *p* = 0.001; stepwise step-down comparisons, adj *p* < 0.05; Fig. 6d), and the species was absent from the Lui valley (Fig. 5b). Known primary vector species were limited to *An. gambiae* s.l. in the wet season (Fig. 5c): two individuals at one dambo transect point within 600 m of the dry season transect point where *An. gambiae* s.l. occurred, with another individual in the adjacent dambo to the north; three in the saturated floodplain edge immediately around Mongu and one in Zambezi floodplain habitat analogous to the Zambezi floodplain location of the dry season specimen. *An. species O/15* occurred predominantly in floodplain habitats, with the highest abundance in the Luena flats followed by the main Zambezi floodplain and significantly lower abundance in floodplain edge and dambo habitats; it was absent from the Lui valley (*H *= 19.169, *df* 4, *p* = 0.001; stepwise step-down comparisons, adj *p* < 0.05; Figs. 5c, 6c). The proportion of unknown anophelines also varied between ecological zones in the wet season, being significantly higher in the Lui valley, Luena flats and dambo habitats than habitats in the Zambezi floodplain and along its edge (*H *= 38.834, *df* 4, *p* < 0.001; stepwise step-down comparisons, adj *p* < 0.05; Fig. 6e).

## Discussion

The combination of a geographically extensive field survey of anopheline larvae, spatially stratified by hydro-ecology, and a genetic identification approach has revealed a complex and dynamic assemblage of potential malaria vectors across Barotseland in western Zambia. Anopheline larvae were found throughout this hydrodynamically complex area and in all ecological zones, but as expected were more widespread and abundant in the wet season (higher proportion of transect points). They were significantly more abundant in the Luena, Lui and dambo ecological zones than in the Zambezi floodplain and floodplain edge in the wet season. Within this widespread anopheline distribution there were substantial differences in abundance and distribution of different species, and differences between wet and dry seasons. A key finding was that in terms of both distribution and abundance, the anopheline population was dominated by secondary vector species. The *An. coustani* group dominated in both wet and dry seasons (55 and 62%, respectively), followed by *An. squamosus*, which was more prevalent in wet than dry season (18 and 8%, respectively), and *An. spp O/15* in the wet season only (5%). These secondary vector species/species groups were widespread across the region, in both wet and dry seasons, although several species (*An. coustani, An. spp O/15*) were more abundant in Zambezi and Luena floodplain habitats. Primary vector species (*An. arabiensis, An. gambiae s.s.* and *An. funestus s.s.*) were relatively rare in both seasons and had very localized distributions consistent between seasons. A group of anopheline individuals that could not be identified to known species comprised 20 and 26% of the dry and wet season population, respectively.

Application of molecular techniques consistent with previous studies ensured that species assignments are comparable with those from eastern Zambia [12] and western Kenya [11]. As in these study areas, we uncovered an unexpected diversity of potential vector species, but with a surprising scarcity of primary vectors (< 2%) among 995 sequenced anopheline larvae. Bias for specific feeding or resting behaviors was avoided by larval sampling and so potentially better represents the whole anopheline community within 1.5 km of villages than adult trapping; although this sampling does not directly demonstrate exposure of the human population to the potential vector species encountered [7], persistent malaria prevalence in the region [16] indicates the presence of substantial numbers of vectors. Given the known diversity of the *Anopheles* genus, with over 140 species in sub-Saharan Africa [5], and the abundance of non-human blood sources across Barotseland (especially livestock [18]), it is expected that non-vector anophelines will be represented in larval sampling. Nonetheless, village-centered surveys encompassing all water body types encountered within known flight range [32] of human blood meals failed to reveal substantial numbers of primary vector species either in the dry season or following the peak of the wet season. Reduced average transect length in the wet season effectively further concentrated sampling on the peridomestic environment, which might have been expected to bias the sample towards more anthropophilic species, yet a smaller proportion of the sample in this season consisted of primary vector taxa.

The primary malaria vector species in Zambia are thought to be *An. arabiensis, An. funestus* s.s. and *An. gambiae* s.s., although detailed entomological studies have been undertaken only comparatively recently [46, 47]. Despite the continued high prevalence of malaria in Barotseland, *An. gambiae* complex and *An. funestus* s.s. represented only 1.7 and 0.1% of anophelines identified by genetic methods in this study. Our result is in contrast to the study by Lobo et al. (2015; [12]), which sampled adult anophelines in villages in eastern Zambia and described a predominance of *An. funestus* s.s. (55% of specimens) followed by *An. arabiensis* and implicated both species in malaria transmission. *An. funestus* s.s. is also thought to dominate transmission in northern Zambia, followed by *An. gambiae* s.s. [48], while in southern Zambia *An. arabiensis* is thought to be the primary vector [47]. However, although *An. funestus* and *An. gambiae* s.l. accounted for 29% and 9%, respectively, of indoor and outdoor collections of adult anophelines in Western Province villages in 2013 [49], they were not the most abundant species in that collection (some of which originated in our study area). All three of these species exhibit large human blood indices in Zambia [50], and although *An. arabiensis* is generally considered to be less anthropophilic than *An. gambiae* s.s. and *An. funestus,* it is more anthropophilic in Zambia than elsewhere in Africa [47].

It is unknown whether the larval community we recorded is a product of the suppression of populations of endophilic and endophagic primary vectors by increased interventions [16], as has been documented elsewhere (e.g. [4, 51, 52]), or is representative of a natural species assemblage of anopheline vectors in Barotseland that has not been historically dominated by primary vector species. The distribution of *An. gambiae* s.l. found in the present study was highly clustered, with eight of ten specimens found within 140 m of each other in the dry season. Sampling after the peak of the subsequent wet season found three of seven *An. gambiae* s.l. associated with the same village, and all specimens across both seasons were closely associated with people (within 600 m of a village). This conforms to the established tendency of *An. gambiae* s.l. to breed in close proximity to human settlements [53, 54] and limit dispersal from these blood sources [32]. The range of aquatic habitats surveyed in our study encompassed streams and large, permanent and heavily vegetated larval habitats typically associated with *An. funestus* and smaller, more ephemeral ones often favored by *An. gambiae* s.l. [48, 55–57], so we do not think biased habitat sampling can explain the low abundance of primary vector species detected. Combined sampling of larval habitat and adults will be needed to further resolve the dynamic relationships among primary and secondary vector populations around villages and across the wider landscape.

*An. coustani* group species comprise up to 65% of surveyed larvae, which supports the limited sampling of adult mosquitoes in Western Province in 2013 that found 60% to be *An. coustani* [49]. The molecularly identified *An. coustani* group in our study contains several closely related species that cannot be further resolved from COI sequences; although some may be separable based on morphology, a number of species remain virtually indistinguishable [58]. In addition, molecular identification of anophelines in eastern Zambia by Lobo et al. has suggested the presence of further morphologically cryptic sibling species, denoted *An. cf. coustani 1* and *2* [12] (one of which may represent *An. crypticus*, the most recently identified cryptic species in the group [59]).

*An. coustani* group was distributed across all ecological zones in this study, although consistently less abundant in floodplain edge habitats. Significantly higher abundance in permanently waterlogged dambo habitats across both seasons reflects the recognized preference of *An. coustani* s.s. for more established water bodies [60]. The species displays a preference for natural vegetated water bodies and an aversion to temporary, non-vegetated pools elsewhere [61], and this is supported by its ubiquitous presence in vegetated habitats [20] across Barotseland in this study. Until recently, *An. coustani* has been considered a secondary vector as it was seen as largely zoophilic [61], but it was recently demonstrated to be the main vector in a village in Madagascar [62] and its secondary vector status is increasingly being reconsidered. *An. coustani* exhibits high levels of anthropophily in some settings [15, 62, 63], practices endophagy as well as (predominantly) exophagy [64–66] and shows a markedly high rate of early biting [62, 63, 67]. It has tested positive for *Plasmodium* infection in Ethiopia [65], Kenya [66] and Madagascar [67]; in Zambia, *An. coustani, An. cf. coustani 1* and *An. cf. coustani 2* have all tested positive [12]. Even low infection rates, in combination with these behavioral traits and locally high abundance [62, 67], may allow *An. coustani* to play a substantive role in malaria transmission.

Among other potential members of the taxon designated as *An. coustani* group in this study, *An. tenebrosus* is assumed not to be a competent vector because of its low parity and long gonotrophic cycle; it has not been detected with malaria parasites [7, 61], and records of presence in Zambia are historical [45]. These traits are also associated with *An. ziemanni*, and although it has occasionally been found to harbor *Plasmodium*, it has historically not been considered a significant vector [7, 61]. Nonetheless, in some areas it demonstrates anthropophily [68, 69], and it may be locally important as a vector in an area of low transmission in Cameroon [70]. Habitat preferences for both these species are also poorly characterized and thought to conform broadly with those of *An. coustani*; both are typically associated with permanent water [61].

*An. squamosus* was also abundant in our larval collections during the wet season. This species is more zoophilic and exophagic than *An. coustani*, although an unexpectedly high degree of anthropophily has been revealed in southern Zambia [63] and Madagascar [71], and *Plasmodium* infection has incriminated the species as a vector in both countries [63, 71]. In Barotseland *An. squamosus* displayed a habitat-specific distribution, predominantly in floodplains and floodplain edge zones. Larvae have been recorded previously from a wide range of habitats, provided they are at least partially vegetated [61], but relatively little is known about this species’ habitat associations.

The third abundant non-primary vector anopheline identified in Barotseland was *An. species O/15*, previously identified in Kenya as a potential sibling species to *An. coustani* [11]. *An. species O/15* is not considered a malarial vector in Kenya (negative for *Plasmodium falciparum*; [11]), and there is no further information on bionomics or larval habitat preferences. In the present study *An. species O/15* constituted 5% of the wet season sample and was significantly more prevalent in floodplain habitats than in other ecological zones.

In eastern Zambia, 39% of 18 delineated anopheline taxa [12], and 53% of 17 taxa in Kenya [11], were designated as ‘unknown’ because of the lack of conclusive similarity to database sequences. While some taxa may represent novel or cryptic species, the lack of an identity may also result from the absence of DNA sequences from known species. In the present study, identifications were based on matches at ≥ 95% similarity to published COI sequences, supported by ITS2 matches using the same threshold, and specimens that failed to yield an above-threshold match (but had consistent below-threshold matches to anopheline sequences, see Additional file 1) were designated as “unknown *Anopheles* species.” Some of these “unknown” individuals may represent further examples of the cryptic species diversity uncovered by recent studies [11, 12], as we know that these individuals do not match to the taxa identified in these studies even though we had high sequence coverage of the same DNA regions; others are likely to be known species that are poorly represented in the NCBI nt database. The occurrence of unidentified anophelines does not affect the key conclusion of this study, because where these individuals are matched (at < 95% similarity) to a sympatrically occurring species, the majority cluster with secondary vector taxa, and we can be confident that they are not primary vectors well represented in the published database. The occurrence of potential further cryptic anopheline species in this and other areas should be taken account of in future studies.

A key remaining question is how representative our comprehensive larval survey is of the distribution of adult *Anopheles* vectors and hence malaria transmission hazard. In some settings, larval densities have been found to be a poor predictor of adult abundance [72], while in others there is very close correlation [73]. If the larval composition is representative, there are significant implications for malaria vector control efforts in the region.

## Conclusions

The diversity of candidate vector species and their high relative abundance observed across diverse hydro-ecosystems indicate a highly adaptable transmission system, resilient to environmental variation and, potentially, interventions that target only part of the vector community. Larval survey results imply that residual transmission of malaria in Barotseland is being mediated predominantly by secondary vector species, whose known tendencies for crepuscular and outdoor biting renders them largely insensitive to prevalent vector control methods.

## Supplementary Information

**Additional file 1: Table S1.** Cytochrome *c* oxidase 1 (COI) sequences for unknown *Anopheles* species. **Table S2:** second internal transcribed spacer region (ITS2) sequences for unknown *Anopheles* species.

**Additional file 2: Figure S1.** Maximum likelihood phylogenetic tree of cytochrome *c* oxidase I (COI) sequences from *Anopheles* larvae sampled in western Zambia.

## Data Availability

The COI and ITS2 sequence datasets generated by and analyzed in the current study are available in the NCBI GenBank nucleotide archive with accession numbers MW167786–MW168163 for COI sequences and MW166490–MW166863 for ITS2 sequences. Additional data on specimens assigned to “unknown *Anopheles* species” category are provided within Additional file 1. A maximum likelihood phylogenetic tree of COI haplotypes is provided in Additional file 2.

## Abbreviations

BLASTn: Basic Local Alignment Search Tool (nucleotide)
NCBI: National Center for Biotechnology Information
