# Supplementary material for: Geographically extensive larval surveys reveal an unexpected scarcity of primary vector mosquitoes in a region of persistent malaria transmission in western Zambia
Source: Parasit Vectors. 2021 Feb 1;14:91. doi: 10.1186/s13071-020-04540-1 (PMC7849156; doi:10.1186/s13071-020-04540-1)
Supplement: Supplementary file 1 — Additional file 1: Table S1. Cytochrome c oxidase 1 (COI) sequences for unknown Anopheles species. Table S2: second internal transcribed spacer region (ITS2) sequences for unknown Anopheles species. [file 13071_2020_4540_MOESM1_ESM.pdf]

Additional File 1

Table S1: Unknown *Anopheles* species: cytochrome *c* oxidase 1 (COI) sequences. Specimens whose COI sequences did not match published sequences with ≥95% identity (on National Center for Biotechnology Information nucleotide database), but whose closest alignments were to *Anopheles* species, were designated "unknown *Anopheles* species". This table provides a breakdown of the species that these sequences aligned with at identity levels below that accepted to represent within-species variation.

**Species category**	**Description** (incl. refs if appropriate)		**Number of haplotypes (individuals); proportion**
Sympatric species (<95% identity)	Anopheline species known to occur in the Afrotropical region	*An. species 1* [1, 2]; *An. species 7* [3]; *An. coustani* group (see designation in text); *An. gambiae* s.l.; *An. squamosus*	21 (22); 13%
Allopatric species (<95% identity)	Anopheline species *not* known to occur in Afrotropical region		30 (114); 70%
Allopatric and sympatric species	Equal similarity (<95%) to both species categories		20 (27); 17%

Table S2: Unknown *Anopheles* species: second Internal Transcribed Spacer (ITS2) region sequences. Specimens whose ITS2 sequences did not match published sequences with ≥95% identity (on National Center for Biotechnology Information nucleotide database), but whose closest alignments were to *Anopheles* species, were designated "unknown *Anopheles* species". This table provides a breakdown of the species that these sequences aligned with at identity levels below that accepted to represent within-species variation.

**Species category**	**Description** (incl. refs if appropriate)		**Number individuals; proportion**
Sympatric species (>95% identity)	Above-threshold match to anopheline species known to occur in Afrotropical region, without COI sequence	*An. pharoensis;*<br>*An. species 16* [3]	3; 6%
Sympatric species (90-95% identity)	Anopheline species known to occur in the Afrotropical region	*An. c.f. coustani 1* [4];<br>*An. pharoensis;*<br>*An. species 16* [3]	10; 19%
Sympatric species (<90% identity)	Anopheline species known to occur in the Afrotropical region	*An. coustani* s.s.*;*<br>*An. mascarensis;*<br>*An. moucheti;*<br>*An. species 6* [3];<br>*An. species 7* [3];<br>*An. species 9* [3];<br>*An. species 11* [3];<br>*An. theileri*	37; 71%
Allopatric species (<95% identity)	Anopheline species *not* known to occur in Afrotropical region		2; 4%

References

1. Bandibabone J, Muhigwa JB, Agramonte NM, Zawadi B, Ombeni L, Corredor-Medina C, et al. Identification of *Anopheles* species in Sud Kivu, Democratic Republic of Congo, using molecular tools. Trans R Soc Trop Med Hyg. 2018;112:405-7; doi: 10.1093/trstmh/try069.
2. Lemma W, Alemu K, Birhanie M, Worku L, Niedbalski J, McDowell MA, et al. *Anopheles cinereus* implicated as a vector of malaria transmission in the highlands of north-west Ethiopia. Parasit Vectors. 2019;12:557; doi: 10.1186/s13071-019-3797-9.
3. St Laurent B, Cooke M, Krishnankutty SM, Asih P, Mueller JD, Kahindi S, et al. Molecular characterization reveals diverse and unknown malaria vectors in the western Kenyan highlands. Am J Trop Med Hyg. 2016;94:327-35; doi: 10.4269/ajtmh.15-0562.
4. Lobo NF, St Laurent B, Sikaala CH, Hamainza B, Chanda J, Chinula D, et al. Unexpected diversity of *Anopheles* species in eastern Zambia: implications for evaluating vector behavior and interventions using molecular tools. Sci Rep-Uk. 2015;5; doi: 10.1038/srep17952.
