# Supplementary material for: Geographically extensive larval surveys reveal an unexpected scarcity of primary vector mosquitoes in a region of persistent malaria transmission in western Zambia
Source: Parasit Vectors. 2021 Feb 1;14:91. doi: 10.1186/s13071-020-04540-1 (PMC7849156; doi:10.1186/s13071-020-04540-1)
Supplement: Supplementary file 2 — Additional file 2: Figure S1. Maximum likelihood phylogenetic tree of cytochrome c oxidase I (COI) sequences from Anopheles larvae sampled in western Zambia. [file 13071_2020_4540_MOESM2_ESM.pdf]

Cross *et al.* Geographically extensive larval surveys reveal an unexpected scarcity of primary vector mosquitoes in a region of persistent malaria transmission in western Zambia

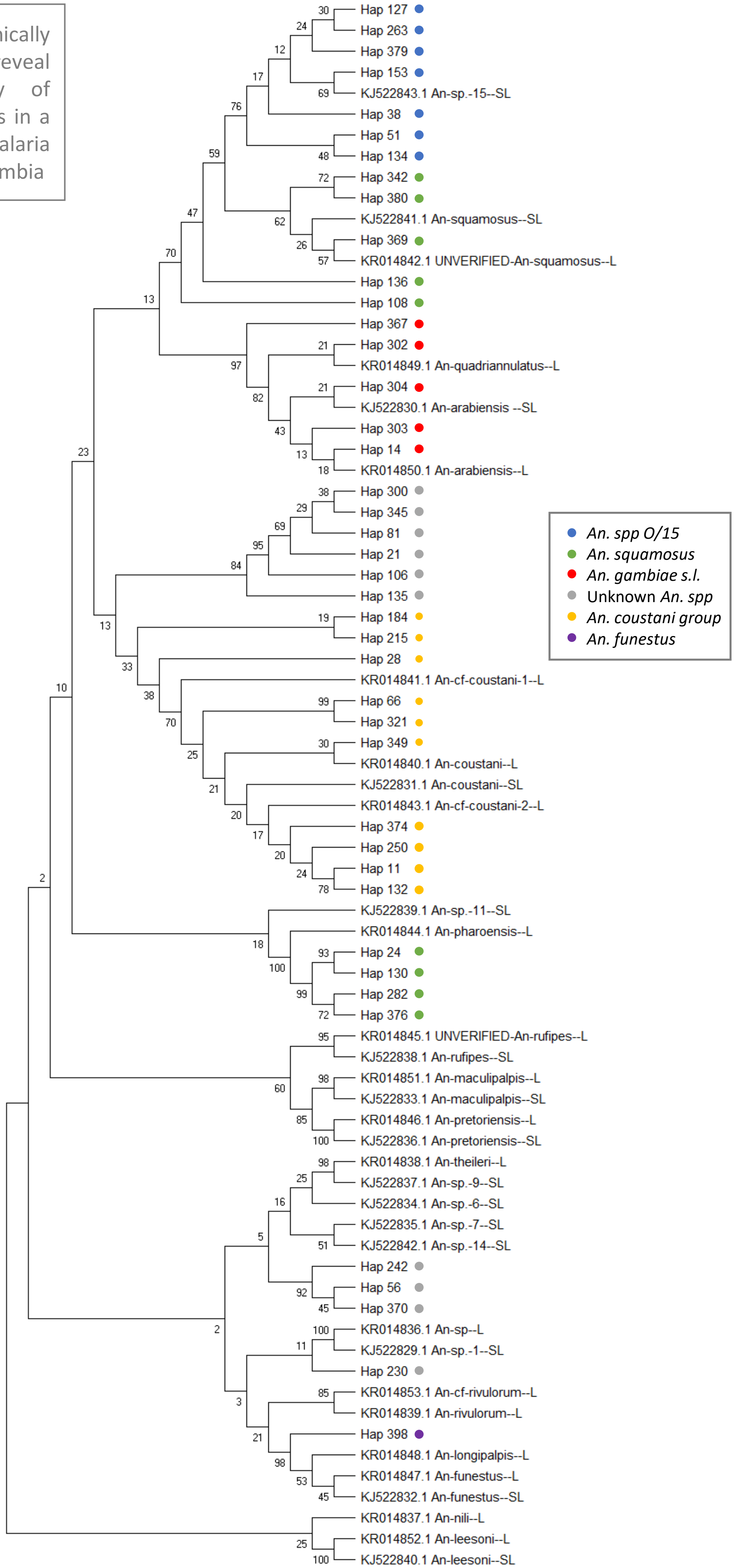

Figure S1: Maximum Likelihood phylogenetic tree of cytochrome *c* oxidase I (COI) sequences from *Anopheles* larvae sampled in western Zambia. Consensus tree constructed in MEGA X from a 318bp fragment of the COI region of mitochondrial DNA, using 100 bootstrap replicates. Sequences from this paper have been labelled with taxon assignment used in this paper. Published reference sequences are labelled with NCBI nt accession number and species ID; suffix denotes source paper. '**--L**' denotes Lobo *et al.* (2015); '**--SL**' denotes St Laurent *et al.* (2016).

St Laurent B, Cooke M, Krishnankutty SM, Asih P, Mueller JD, Kahindi S, et al. Molecular characterization reveals diverse and unknown malaria vectors in the western Kenyan highlands. Am J Trop Med Hyg. 2016;94:327-35; doi: 10.4269/ajtmh.15-0562.

Lobo NF, St Laurent B, Sikaala CH, Hamainza B, Chanda J, Chinula D, et al. Unexpected diversity of *Anopheles* species in eastern Zambia: implications for evaluating vector behavior and interventions using molecular tools. Sci Rep-Uk. 2015;5; doi: 10.1038/srep17952.
